# Upgrading the Quality of Recycled Aggregates from Construction and Demolition Waste by Using a Novel Brick Separation and Surface Treatment Method

**DOI:** 10.3390/ma13132893

**Published:** 2020-06-27

**Authors:** Kui Hu, Yujing Chen, Caihua Yu, Dong Xu, Shihao Cao, Rui Pang

**Affiliations:** 1 College of Civil Engineering, Henan University of Technology, Zhengzhou 450001, China; mailhukui@haut.edu.cn (K.H.); mailhk87@gmail.com (C.Y.); shcao@haut.edu.cn (S.C.); 2 Department of Civil and Environmental Engineering, Michigan Technological University, 1400 Townsend Drive, Houghton, MI 49931, USA; 3 Shenzhen Municipal Design and Research Institute Co., Ltd., Shenzhen 518029, China; feskio2007@gmail.com

**Keywords:** construction and demolition waste, recycled aggregates, brick separation, surface treatment

## Abstract

Mixed recycled aggregates (MRA) from construction and demolition waste (CDW) with high-purity and environmental performance are required for highway construction application in base layer and precast concrete curbs. The main problematic constituents that reduce the quality level of the recycled aggregates applications are brick components, flaky particles, and attached mortar, which make up a large proportion of CDW in some countries. This paper studies the potential of brick separation technology based on shape characteristics in order to increase the recycled concrete aggregates (RCA) purity for MRA quality improvement. MRA after purification was also processed with surface treatment experiment by rotating in a cylinder to improve the shape characteristics and to remove the attached mortar. The purity, strength property, densities, water absorption ratio, shape index, and mortar removal ratio of MRA were studied before and after the use of the brick separation and surface treatment proposed in this study. Finally, the recycled aggregates upgradation solution was adopted in a stationary recycling plant designed for a length of 113 km highway construction. The properties of CDW mixed concrete for precast curbs manufacturing were conducted. The results indicate that problematic fractions (brick components, particle shape, and surface weakness) in the MRA were significantly reduced by using brick separation and surface treatment solution. Above all, it is very important that the proposed brick separation method was verified to be practically adopted in CDW recycling plant for highway base layer construction and concrete curbs manufacturing at a low cost.

## 1. Introduction

Construction and demolition waste (CDW) represent one of the largest waste streams worldwide because of continuous industrial development, the demolition of infrastructure, and house-building activities. China produces approximately 30% of the municipal solid waste in the world, of which approximately 40% is the CDW materials. About 100 million tons of construction and demolition waste is generated from the new buildings, whereas, about 200 million tons of CDW is produced by demolition of old buildings in Shenzhen, China [1]. Furthermore, with the progressive depletion of natural aggregates (NA) for infrastructure construction and the growing awareness of sustainable waste management, the demand for CDW recycling in civil engineering projects is expected to increase [2].

A large proportion of CDW was used or recycled at construction sites for low-grade unbound applications such as embankments, foundations, and landfills [3]. For road construction applications, the largest recycled proportions of CDW are the stony fractions. A high recycling rate of mixed fractions of concrete, brick, mortar, and tiles was achieved in some countries, but CDW is mostly reused in low-grade applications such as nonstructural concrete mixtures, embankments, subbase, and road leveling [4,5]. The market for low-grade CDW reuse, however, is gradually becoming increasingly limited due to the low quality of the recycled aggregates [6]. Therefore, a shift toward recycled aggregates with high-grade purity, strength, stability, and flaky structure is currently investigated and promoted. Bricks and concrete structures are the primary building types in China and other countries [7]. Typical recycled components for civil engineering reuse in CDW are concrete and brick, which are generally believed to be a substitute for virgin natural aggregates in civil engineering [8]. However, the inclusion of recycled brick as an aggregates decreases the performance of the mixed recycled aggregates (MRA) dramatically because of its inherent porous nature as an aggregate The brick constituents mixed in recycled aggregates are a major quality problem due to its relatively large water absorption and low strength resistance. Therefore, the use of recycled brick as a partial aggregates substitute should be confined to low-volume replacement levels for pavement mixtures and concrete manufacturing [9].

Currently, CDW recycling technologies consist of a vibratory feeder (primary screening), impact crusher, magnetic belt conveyor, secondary screening, and air blower. In order to produce high-quality CDW recycled aggregates, more rigorous separation and sorting techniques are needed to meet the required purity, soundness, water absorption ratio, compressive strength, etc. [10,11]. Advanced automated sorting techniques by gravity concentration, color, X-ray, near-infrared, and spectral parameters have been successfully researched and developed to upgrade the quality of recycled aggregates [12,13,14]. In the field of CDW recycling, the preliminary tests of brick/concrete automatic separation techniques based on gravity concentration were carried out on a laboratory scale [15,16]. Because CDW recycling for concrete production and pavement construction need a large volume of recycled aggregates, further brick separation techniques are essential to be adopted in a CDW recycling plant.

On the other hand, due to the large water absorption and weak bond strength with new matrix, the mortar attached on the aggregates was another factor which degraded the quality of recycled aggregates [17]. Both the micro-porosity in cement paste and the mesoporosity between particles increase the water absorption of RCA [18]. In order to remove the attached mortar from RCA, an acid-soaking process using a sodium silicate solution was carried out in the laboratory. The microbial carbonate precipitation and specific surface treatment methods can increase the density and reduce the water absorption of RCA [19,20]. However, the above listed techniques used to strengthen RCA are all carried out on a laboratory scale, however, further research technologies which are flexible for CDW recycling plant adoption are required.

Most of these methods are energy intensive and are not practical for CDW recycling plant because of their high operation costs. This paper reports on an investigation into concrete/brick separation solutions on a large processing scale based on the brick and concrete shape characteristics in order to improve the purity of MRA. Another objective of the current study is to assess and discuss the performance of brick/concrete separation and surface treatment solutions which provided a purified MRA with a lower proportion of brick constituents and attached mortar.

## 2. Materials and Methods

### 2.1. Description of Test Section Materials

**Figure 1:**
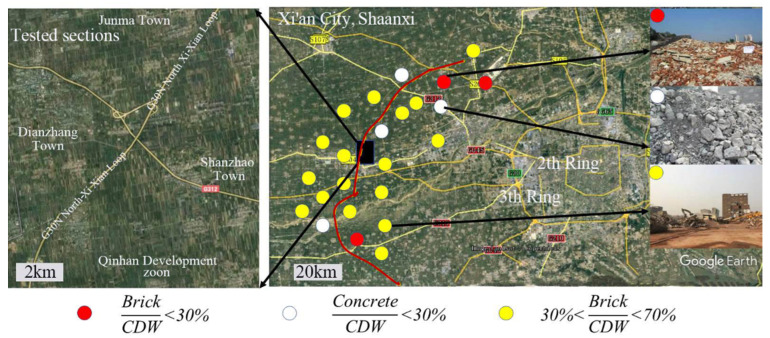
Construction and demolition waste (CDW) site distribution along the highway to be built.

As shown in Figure 1, the CDW raw material constituents were investigated along the highway that was planned to be built in Xi’an city, China. The route lies in a representative suburb area with many old buildings to be demolished in demand of city’s development and expansion. The total design mileage is 113 km as one part of the fourth ring expressway in the city, named as G30N Xi-Xian Loop in Figure 1. Representative 30 kg of CDW materials were collected for each sample. As marked on the map, CDW along the highway we investigated was classified as brick/CDW < 30%, concrete/CDW < 30% and others. As investigated the samples from 24 CDW sites, 21 samples with brick proportion of more than 30 wt.% were taken up 87.5%.

The predominant constituents of the CDW raw materials were brick, concrete, clay, and mortar. Table 1 shows the chemical compositions of the above constituents and lime stone aggregates. The constituents of brick and clay showed a close chemical composition, primarily consisting of more than 80% by silica and aluminum oxides as well as a small proportion of other oxides (Fe_2_O_3_ and MgO). The chemical compositions of concrete and mortar primarily consisted of silica, aluminum oxides, and calcium oxide (55.5 wt.%, 50.84 wt.% and 19.81 wt.%, respectively). Natural limestone and representative recycled aggregates showed a large difference in chemical compositions as presented.

### 2.2. Brick Separation Technology

**Figure 2:**
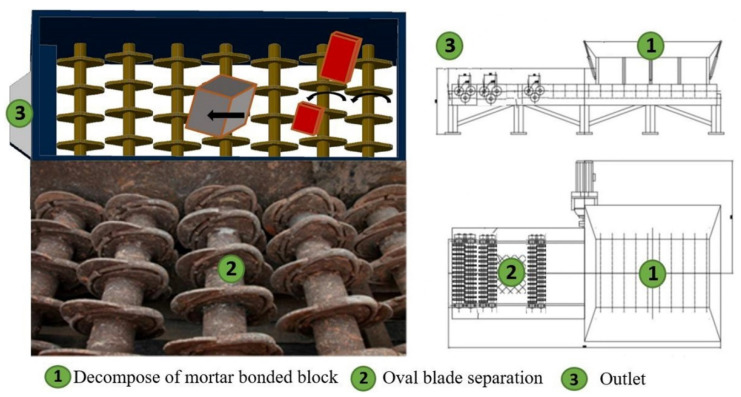
Functional principle illustration of brick separation technology based on shape characteristics.

**Figure 3:**
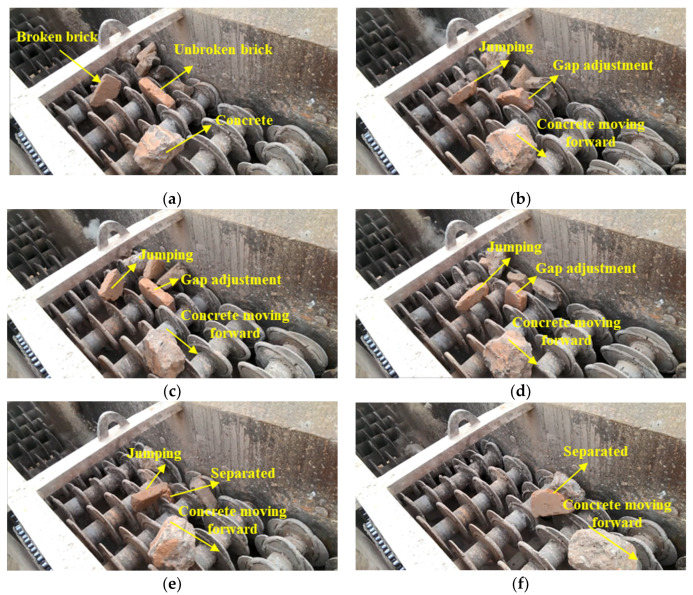
Video snapshots of the brick separation process: (**a**) CDW materials feeding; (**b**) Broken brick jumping between axle; (**c**) Bulk concrete move forward; (**d**) Unbroken brick pass the axle gap; (**e**) Unbroken brick separated; (**f**) Broken brick separated.

It is generally known that properties such as strength, air voids, water absorption ratio, soundness, shape and surface roughness have a huge difference between recycled brick and concrete. Separating brick is very challenging due to the multi-constituents and multi-size raw materials in CDW raw materials. Another important consideration is the processing capacity which should be suitable for CDW recycling plant as well as in a low cost. Some advanced automated CDW sorting techniques were based on the different indexes of density and electrical conductivity, or magnetic susceptibility, by using the methods of jigging, air blowing, near-infrared spectrum, or visible color separation technologies. This study focuses on the use of a separation method based on shape characteristics aimed at separating the brick constituents of CDW raw materials. Figure 2 and Figure 3 shows the innovation machine design and operation process for brick separation technique proposed in this study and described as follows.

(1) Bricks block decomposition. As shown in Figure 2, CDW raw materials are lifted by a loader and fed into feedrollers ①, where materials can be discharged at a controlled speed. The bulks of the bonded bricks were decomposed into pieces of brick after being squeezed by the feedrollers ①. Bricks were easily separated into pieces by splitting force due to fragile mortar bonding strength. The feedrollers ① were designed to break the bonded mortar by providing proper shearing stress as well as a controlled CDW outlet speed to the separation area ②. Brick pieces and concrete (red and gray cuboid in Figure 2) were evenly fed onto the oval blade separation area ②, where brick pieces passed through the roller blade gaps. As shown in Figure 2, concrete moved in the direction to outlet ③.

(2) Cuboid brick passed through the gap during the jumping and rotating process. The size of bricks in China was in accordance with a standard of 240 mm × 115 mm × 53 mm. The gap between two adjacent axles was designed as 118 (± 2.5) mm × 55 (± 1) mm as shown in Figure 3. The point is the smallest cross section of broken and unbroken bricks would exactly pass through the 118 (± 2.5) mm × 55 (± 1) mm gaps on the oval blade separation area, as shown in Figure 2. The CDW materials fed on the separation area consist of broken bricks, unbroken bricks and the concrete bulks, as shown in Figure 3a. Special oval-shaped blades were designed and welded on each axle to provide proper force which pushes the jumping brick into the the separation area as shown in Figure 3b. Another important technology is the design of adjacent axles rotating in the opposite direction at the same time. The unbroken bricks roll over from Figure 3a–d several times until the cross section of 115 mm × 53 mm approaches the gap between the blades, as shown in Figure 3d. The concrete bulk in Figure 3c keeps moving to the outlet because of its oversize for the axle gaps and overall inclination of the machine. After jumping many times on the separation area, the unbroken brick and broken brick were eventually separated in Figure 3e,f. Meanwhile, the concrete bulk moves forward to the outlet in Figure 3f.

### 2.3. Surface Treatment

**Figure 4:**
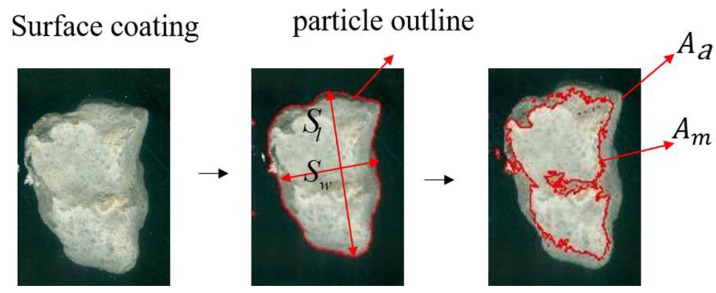
Illustration of shape characteristic indexes.

Elongated and flaky particles encompass a large proportion of the recycled aggregates in the crushing process of CDW. One surface treatment method with aggregates rotating and impacting in a cylinder was proposed to improve the shape and surface coating of recycled concrete aggregates. RCA samples were coated with white alkyd to obtain a clear outline by a high degree of color contrast as shown in Figure 4. Two indexes, N and R, were put proposed to evaluate the shape characteristics before and after surface treatment. The elongated and flaky characteristics are defined by Equation (1), whereas, N values larger than 3 are believed to be unsuitable for pavement materials. The closer the shape is to a circle, the closer the N value is to 1.

(1) $$N=\frac{S_{l}}{S_{w}}$$

where $S_{l}$ is the maximum length of the particle and $S_{w}$ is the minimum width of the particle.

The shape characteristic of roundness is calculated by Equation (2). The closer the shape is to roundness, the closer the R value is to 1.

(2) $$R=\frac{l^{2}}{4\pi A}$$

where *l* is the length of the particle outline and *A* is the area of the particle outline, as indicated shown in Figure 4.

**Figure 5:**
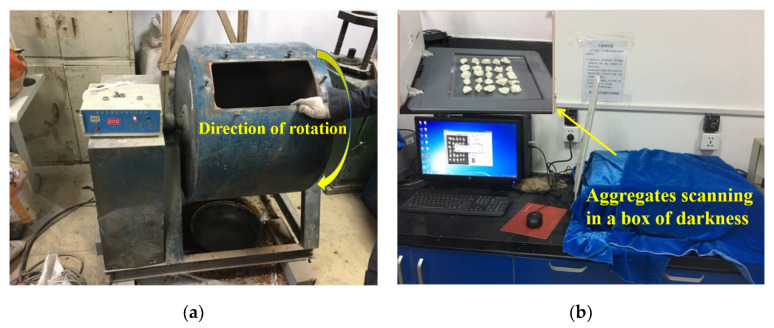
Surface treatment experiment in laboratory: (**a**) Los angles abrasion machine; (**b**) parallel light scanning machine.

It is generally accepted that the properties of recycled aggregates degradation are low due to surface mortar coating and weakness after years of usage in bulk concrete. RCA were tested in a cylinder with an inner diameter of 710 mm and a rotation speed of 30 revolutions/min, as shown in Figure 5a. The aggregates would drop to the bottom when rotated on the top point of the cylinder during operation. The removal ratio of surface mortar was measured by the white alkyd coating on the aggregates under the chosen rotation cycles of 20, 70, 150, and 350. The surface shape characteristics were observed in a dark box to capture their images as shown in Figure 5b. Composed of a scanning machine, a wooden shell, and light shading cloth, the dark box was self-made that could provide parallel light to obtain a better aggregate surface and its outline. The mean mortar removal area ratio P is used to evaluate the quality improvement for surface weakness removal and is given by

(3) $$P=1-\frac{A_{m}}{A_{a}}$$

where *A_a_* is the area of the aggregates particle outline and *A_m_* is the area of white coating after surface weakness removal, as shown in Figure 4.

### 2.4. Preparing Brick Aggregates Mixed Concrete

The reference brick aggregates mixed concrete was designed with a target compressive strength of 30 MPa. To study the effect of brick mixing on the basic properties of concrete and the feasibility for concrete manufacturing, mixtures were classified into 11 groups with one gradation design. As shown in Table 2, the 11 groups of mixtures were designed with brick aggregates contents of 5 wt.%, 8 wt.%, 12 wt.%, 15 wt.%, 17 wt.%, 23 wt.%, 31 wt.%, 41 wt.%, 51 wt.%, 62 wt.%, and 74 wt.%. Composed by 50 wt.% recycled brick aggregates and 50 wt.%, RCA were mixed in the concrete samples with constituents of 36 wt.%, 35 wt.%, 35 wt.%, 35 wt.%, 34 wt.%, 33 wt.%, 33 wt.%, 33 wt.%, 31 wt.%, 30 wt.%, and 30 wt.%. The cube samples were cured for up to 3 days, 7 days, 14 days, 28 days, and 90 days for the compressive strength test (ASTM C 39) and splitting tensile test (ASTM C 78).

## 3. Results

### 3.1. MRA Quality Evaluation by Brick Separation Treatment

**Figure 6:**
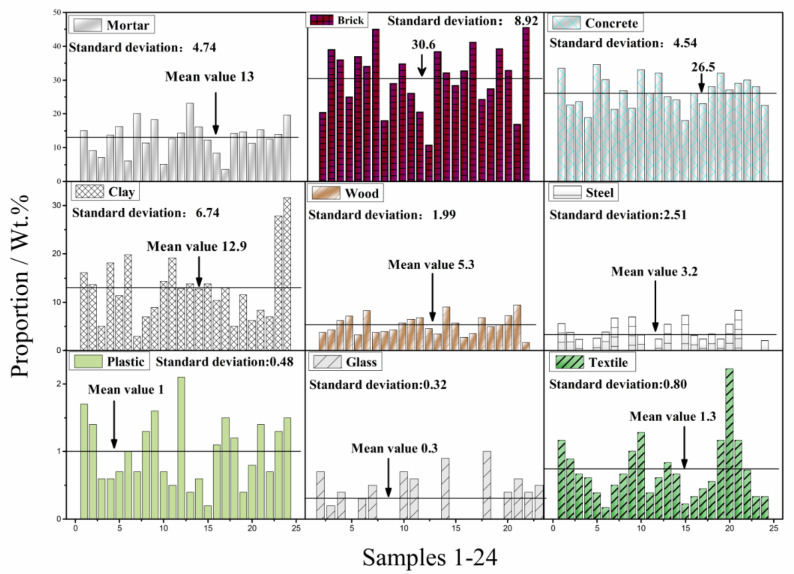
Constituents of CDW along the highway test section.

As shown in Figure 6, the constituents of the twenty-four samples collected in the suburbs of the city as shown in Figure 1 were classified manually, and the average weight proportions were provided. The predominant constituents of the CDW were brick, concrete, clay and mortar, with mean values of 30.6%, 26.5%, 12.9%, and 13%, respectively. A low pre-classification level and a high proportion of the brick fraction are the main differences in CDW constituents between China and some other countries such as the USA, Japan, and Germany in the past decade [21]. The results from the twenty-four samples were the typical CDW constituents for most cities in China and some other countries in Asia and European [22].

**Figure 7:**
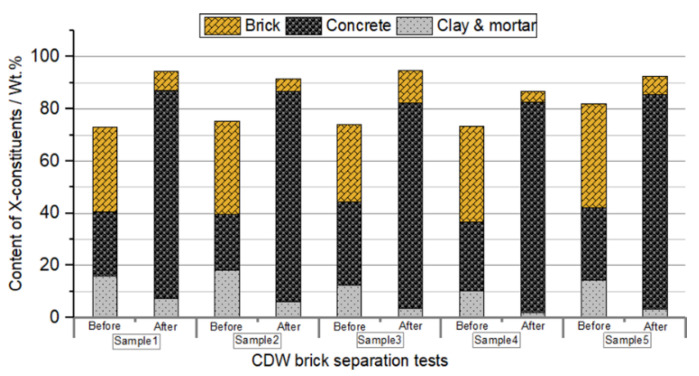
Constituents of the mixed recycled aggregates (MRA) before and after the brick separation test.

Figure 7 represents the constituent proportions for the five samples of CDW raw materials before and after being subjected to the brick separation test based on shape characteristics. Four of the five samples revealed brick proportions lower than 10% after being separated. The maximum reduction of brick constituents was achieved in sample 4 which varied from 36.5% to 4.3%, while the minimum reduction in performance was obtained in sample 3 with a 12.4% brick proportion after being separated. For most of the samples, the maximum concrete proportion makes up to more than 80%, and the clay and mortar proportion is reduced to less than 6% after the brick separation treatment proposed in this research.

By using color, gravity concentration, color, and X-ray separation techniques in the present literature, the maximum concrete proportion can be obtained larger than 90 wt.% [12,13,15]. Due to the proposed method and its operation cost, the above-mentioned techniques and the devices only were reported on a laboratory scale. To promote the sustainable development in CDW recycling, operation cost is one important complication for modern technologies to be adopted by CDW recycling plant. At the same time, the separation device should have a production ability cooperating with the crusher and screening machine in one production line. For this reason, the new brick separation technology and device developed in this study considered both quality upgradation of recycled aggregates and its practicality in production application. Therefore, comparing with the reported brick separation technologies in the present literature, the separation technology proposed in this study behaved at lower cost as well as can be allied in the CDW recycling plant on a larger scale.

**Figure 8:**
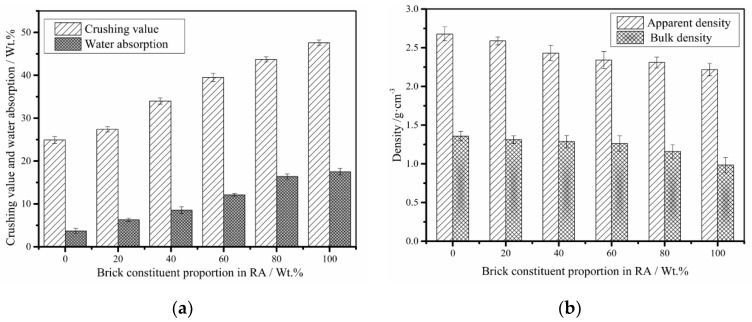
Indexes of MRA with brick proportion variations (AASHTO T 19M/T 19): (**a**) Crushing value and water absorption; (**b**) Apparent density and bulk density.

**Figure 9:**
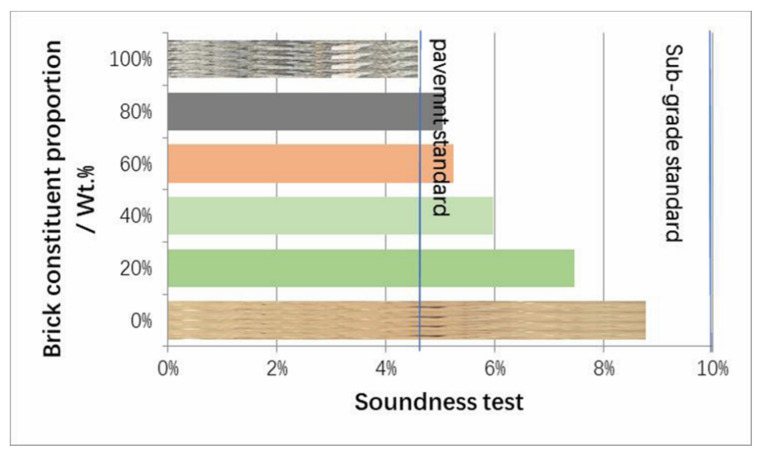
Soundness test of the MRA with brick proportion variations (ASTM C88).

Figure 8 and Figure 9 provide basic indexes for building materials of MRA, such as crushing value (BS EN 1097-2; Crushing value is used to measure the ability of aggregates to resist crushing under load of 400 Kn in 10 min), apparent density, bulk density, and soundness for aggregates, with variation in the brick constituent proportion. Soundness test is the one method to test the durability performance of pavement aggregates (ASTM C88): the aggregates were subjected to five cycles of soaking and drying with saturated sodium sulfate. The soundness index is calculated by Equations (4) and (5).

(4) $$Q_{i}=\frac{m_{i}-m_{i}^{\prime }}{m_{i}}\times 100$$

(5) $$Q=\frac{\sum^{\;}m_{i}Q_{i}}{\sum^{\;}m_{i}}$$

where *i* is the aggregates size distribution level, *m_i_* is the mass of *i*-level aggregates in dry condition before testing, *Q_i_* is the mass loss percentage of *i*-level aggregates, and *Q* is the soundness index.

Obviously, with the decrease in the brick proportion of MRA, the above indexes for MRA properties were prominently improved. For the indexes of apparent density and bulk density, the difference between the recycled concrete and recycled bricks ranged from approximately 0.2 to 0.3 g/cm^3^. The water absorption value of recycled concrete is 3.7%, which is approximately 1% higher than that of natural aggregates. However, the water absorption value of the recycled brick reaches up to 17.5% which is much beyond the standards of pavement and concrete aggregates. This is mainly due to the porous microstructures of brick made from fire clay [20,23]. High porosity of brick aggregates can promote high water consumption during mixing and thus largely increase the water and binder ratio in mixture designs.

It was reported that the decrease in the crushing value in compressive strength was due to the fractions with crushing of weakness and porous characteristics [24]. The high porosity of brick is an important factor leading to the soundness reduction in the MRA, as shown in Figure 9. Because of the high porosity of MRA mixed with brick, the durability, and strength properties of the concrete mixture containing MRA were reduced. Although the brick proportion degraded the MRA quality, the results showed that the brick proportion lower than 20% exhibited no significant negative effect on the above aggregates indexes. This brick separation equipment can be used at a relatively low cost as well as replace the feeder machine (or presorting machine) in order to recyle CDW especially on a larger scale.

Thus, brick separation from CDW by shape characteristic appeared to be an alternative treatment method for CDW recycling. Compared with color separation and density separation, the brick separation method proposed in this study produced an MRA with a relatively low purity of RCA, indicating that brick separation by shape characteristic is less selective than that of color and density separation.

### 3.2. RCA Quality Evaluation by Surface Treatment

After brick separation from CDW materials, approximately more than 85 wt.% RCA purity was obtained, as shown in Figure 6. The mortar coated on gravel and surface weakness were the main factors for the quality degradation of MRA. In this study, one surface treatment method of MRA after brick separation was proposed by using a rotating compactor for shape and surface characteristic optimization, as shown in Figure 5.

**Figure 10:**
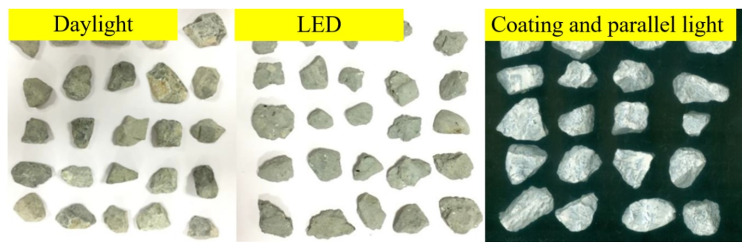
Surface image capture methods for recycled aggregates.

To obtain the shape characteristics and the amount of mortar coating, the aggregates images were captured and compared in three light sources: daylight, LED, parallel light in a dark box. As shown in Figure 10, the images captured in daylight exhibited shadows and unclear outlines. The LED obtained an aggregates outline without shadows but the image contrast ratio was limited.

**Figure 11:**
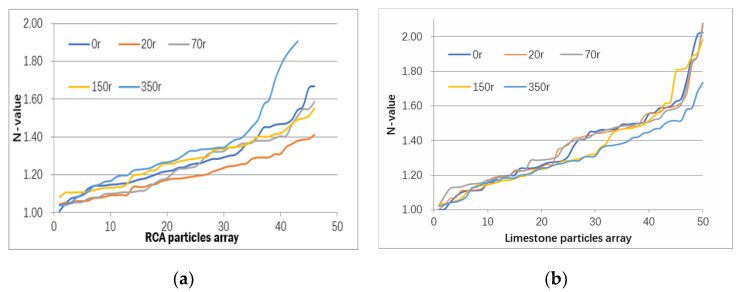
N-value variation of RCA and nature aggregates under surface treatment: (**a**) RCA particles; (**b**) Limestone particles.

To obtain the aggregates images with clear outlines and mortar coatings, a surface coating and shadow-less scanning method was proposed, as shown in Figure 10. The aggregates to be tested were coated with white alkyd to obtain a high contrast with the background. By using white alkyd coated on the aggregates, the variation in mortar loss during the surface treatment test can be observed and calculated. After that, the aggregates were scanned in a self-designed black box covered on the scanner with a parallel light as shown in Figure 5b. The RCA exhibited a clearer surface and outline by using this method when compared with those of the image capture methods in daylight and LED. As shown in Figure 11, the variation in the weakness removal ratio p (Equation (3)) during surface treatment was calculated by using MATLAB™ image data analysis.

Parameter N in Equation (1) is the index used to evaluate the elongated and flaky characteristics of the aggregates. Figure 11 provided the N value variation of 50 RCA and natural aggregates samples, which were observed by rotating 0, 20, 70, 150 and 350 cycles in a cylinder without steel balls inside. The cylinder used the equipment as in ASTM C 535. Generally, the N values of the RCA and limestone are within a range of 1.0 to 2.0 during the surface treatment. RCA were more susceptible to the surface treatment method than the limestone particles.

During the first 20 cycles of surface treatment for RCA, the average N value was reduced from 1.27 to 1.19. The lowest value of N was obtained by 20 cycles of surface treatment, which is also the ideal shape index of elongated and flaky characteristics. As the treatment cycles were increased to 70, 150, and 350 cycles, the mean N value of the RCA particles increased to 1.24, 1.27, and 1.32 after a decrease from 0 to 20 cycles of treatment. This indicated that the N value could be improved in a proper treatment range around 20 cycles in this test.

On the other hand, the N value for the natural aggregates was slightly decreased with treatment cycles in general. The lowest N value close to 1 is obtained for aggregates that are rotated in the cylinder for 350 revolutions. It is thus presumed that this surface treatment method has no significant improvement for natural aggregates but can be effective to upgrade RCA quality.

**Figure 12:**
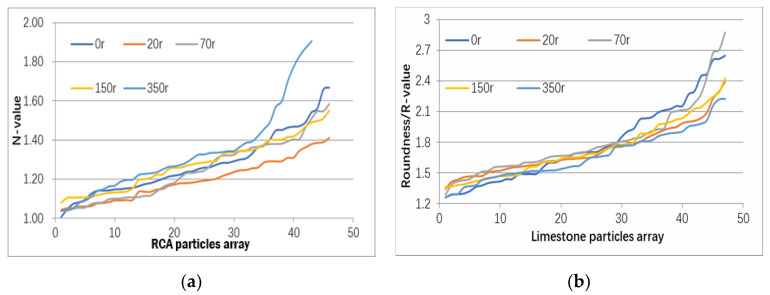
R-value variation of (**a**) RCA and (**b**) nature aggregates under surface treatment.

Parameter R in Equation (2) is the index used to evaluate roundness characteristics of aggregates. Figure 12 shows the R values of 50 RCA and natural aggregate samples under rotating in the cylinder under 0, 20, 70, 150, and 350 revolutions, aiming to improve the shape characteristics by the surface treatment method. Generally, it is indicated that the treatment cycles are effectively improved the R index of RCA. The R values of the original 40 RCA in the samples ranging from 1.5 to 6.5 gradually reduced to the range from 1.1 to 2.0, which was an ideal roundness characteristic for most of building aggregates [25]. The surface treatment method for the R value was optimized at 70 revolutions with a low level and small range distribution. On the other hand, the RCA exhibiting a lower R value was more susceptible to the surface treatment method than natural aggregates. It can be seen that higher level of rotation in the surface treatment results in a decrease in the R value and thus upgrades the aggregate quality.

**Figure 13:**
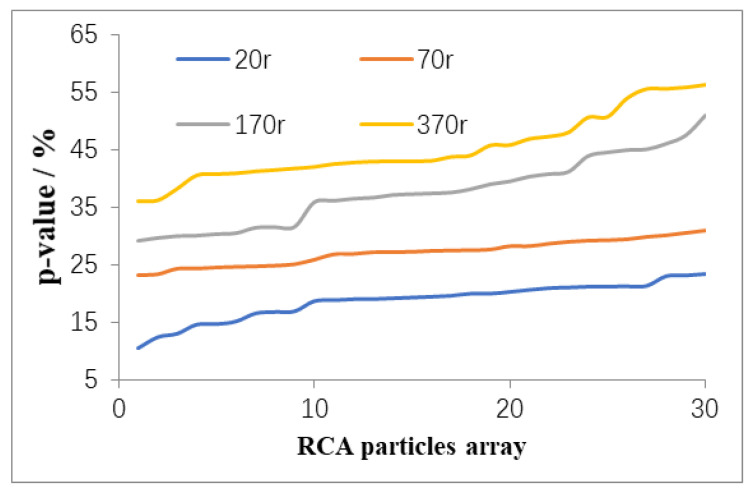
Variation in *p*-value under surface treatment.

Parameter P in Equation (3) is the index used to evaluate the mortar removal ratio by using the surface treatment method. Figure 13 shows the *p* values of 30 RCA samples under rotation in the cylinder at 20, 70, 170, and 370 revolutions. It is shown that the *p* value increases gradually as the number of revolutions increases from 20 to 370. The *p* value after 70 cycles of treatment obtained a small range of 25% to 30%. Most of the mortar and weak components of the RCA were removed during the treatment process from 0 to 70 cycles. After the first period of 0 to 70 cycles, the original particles of the RCA began to breakdown into finer particles.

After brick separation from the CDW materials, the surface treatment of the RCA generates a product with a lower content of flaky particles, surface mortar, and weak fractions. Considering the RCA content after separation of higher than 85% in most cases (Figure 7), the quality of MRA from CDW can be improved or significantly upgraded by surface treatment. Regarding surface treatment by rotation, the removal of MRA weakness and attached mortar phase represented 25–30% of the total samples. The quality improvement of RCA is notable, as indicated by Figure 12 and Figure 13. Considering optimized rotating treatment method at 70 revolutions, the quality of the RCA improved significantly. Compared with the carbonation of mortars and alkali activation treatment methods [17,26], surface treatment by rotation appears to be an alternative for CDW recycling, with the advantages of upgrading the RCA properties at a low cost and more applicable for CDW recycling.

### 3.3. Field Evaluation of Upgraded MRA Production for Highway Construction

**Figure 14:**
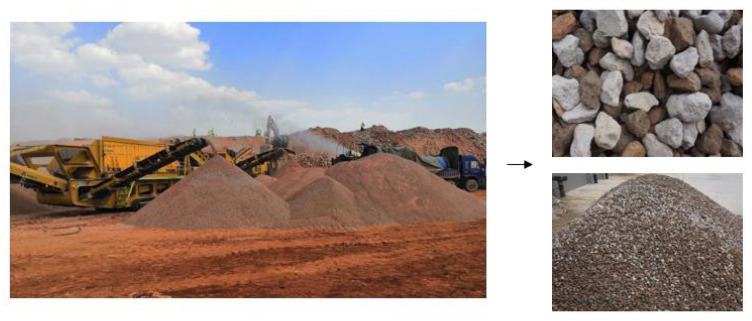
Mobile plant of CDW and MRA product.

**Figure 15:**
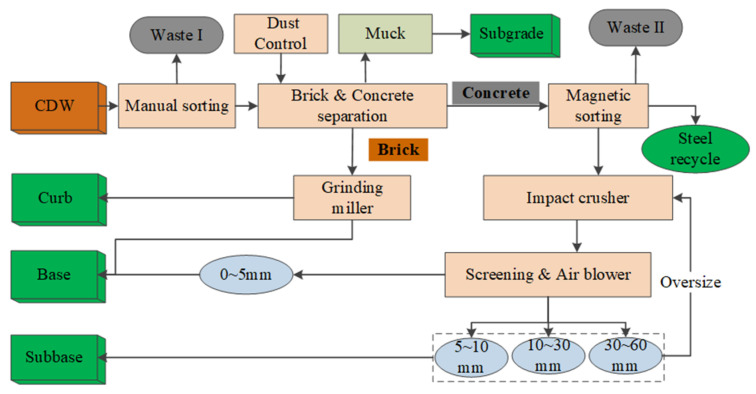
Schematic diagrams of a stationary plant equipped with an upgrading solution.

To produce aggregates for highway construction presented in Figure 1, a mobile plant (as shown in Figure 14) and a stationary plant (as shown in Figure 15) were adopted for CDW recycling on a larger scale. Approximately 60 million tons of CDW along the highway was recycled as a substitute materials mainly for foundation treatment, small precast concrete curbs, subbase layer mixtures for a 113 km and six lanes of highway construction.

As shown in Figure 14, the mobile plant was equipped with an impact crusher, magnetic conveyor, light components removal, and aggregates sieving for a maximum particle size of 80 mm. The compositions of recycled aggregates exhibited heterogeneity and varied depending on the CDW raw materials. The portable processing was suitable for scattered CDW sites distributed far away from the construction site as shown in Figure 1. Figure 15 shows a stationary plant for the CDW recycling process used in this project for large-scale upgraded MRA production by using brick separation and surface treatment technology adopted in this study.

**Figure 16:**
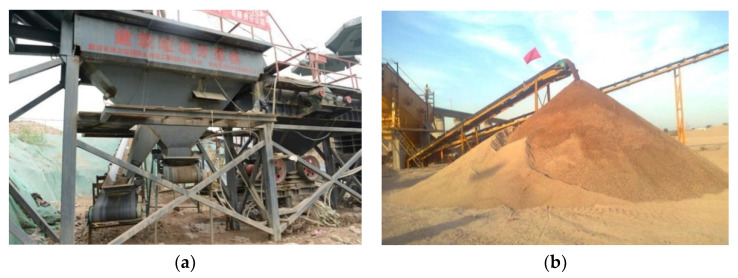
Brick separation technology and upgraded recycled aggregates production: (**a**) Brick separation device; (**b**) Purified recycled aggregate.

First, some raw materials unsuitable for crusher, such as oversize wood, stone, steel, textile, and so on, were manually sorted before the loader feed CDW materials into brick separation device. Second, brick constituents were separated from CDW by using the proposed separation technology in this study, as shown in Figure 16a.

Second, the brick constituents were then fed into grinding miller and recycled for curb and base layer mixtures. At the same time, the brick separation device screens 0–50 mm size CDW materials used for foundation treatment of highway as shown in Figure 15 or Figure 16b.

Then, concrete constituents were processed with magnetic sorting machine and the impact crusher to sort the steel inside and reduce their size for curb and base layer mixture application. A cone crusher with function of surface treatment was also adopted for RCA processing as show in Figure 15.

Finally, the investment cost of the brick separation equipment (including installation and conveyors) varies, depending on the production capacity, between approximately ¥0.4 million Chinese Yuan ($ 60,000) to roughly ¥ 0.6 million Chinese Yuan ($ 82,000). A treatment capacity of 50–70 tons/hour was accomplished in this highway construction project at a cost of 4–6 ¥/ton for CDW recycling.

As shown in Figure 15, the materials after brick separation were processed by magnetic sorting, impact crushing, screening and air separation. In this process, steel in the concrete was collected, 0–5 mm fine particles were recycled in the subbase and aggregates larger than 60 mm were returned to the impact crusher. Finally, a cone crusher with surface treatment function was adopted to improve the shape characteristic and to remove the attached mortar.

**Figure 17:**
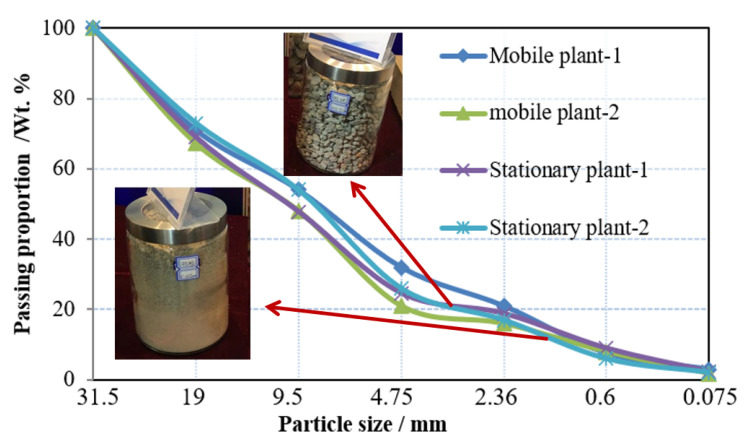
Particle size distribution of crushed materials for mobile plant and stationary plant.

**Figure 18:**
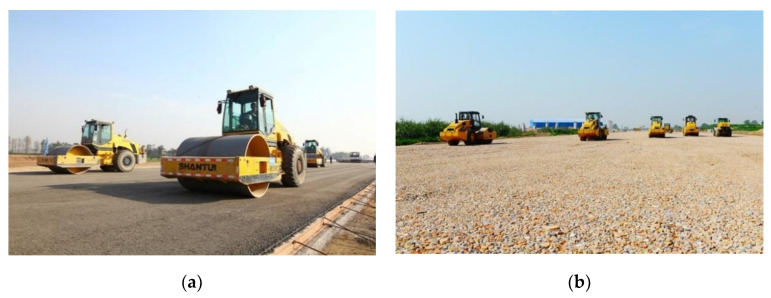
Upgraded recycled aggregates used in highway construction: (**a**) Base constructed using purified MRA; (**b**) Sub-base constructed by brick mixed MRA.

Figure 17 shows the particle size distribution of crushed materials for mobile and stationary plant. The upgraded recycled materials were obtained with less impurities, better shape characteristic, and uniformity [9,27]. With a replacement ratio ranging from 30 wt.% to 35 wt.%, the upgraded recycled materials produced by stationary plant (Figure 15) were used for base construction and small precast concrete curbs of highway in Figure 1 (Figure 18a). The low-grade high brick proportion and the products from mobile plant were used for subbase construction (Figure 18b) and other low-grade application. According to the current engineering standard for pavement materials (JTG/T F20-2015) and recycled concrete materials (GB/T 25177-2010) in China, the quality of recycled aggregates was upgraded from level-3 to level-2 for recycled concrete materials as well as from subbase material to base material for highway construction.

The quality of recycled aggregates was upgraded for concrete proportion and surface treatment; Currently, there are no reports or trials for brick separation on a large scale for pavement construction. A 113 Km highway was constructed by using the recycled materials from CDW. It is the largest scale for CDW recycling project as reported in currently literature. The practical solution proposed in this study played a vital role to develop the CDW recycling for highway construction on a larger scale. According to the estimation data from this project, more than 6.5 million tons of CDW recycled materials was used, about 1870 acres of land with CDW occupied was cleared, saving more than 32 thousand tons of coal consumption, reduced 3.6 million m^3^ of total CO_2_ emission. For highway construction for the use from CDW recycling plant, brick separation and surface treatment method proposed in this study proved to be a practical and promising technology.

### 3.4. Small Precast Concrete Curbs

**Figure 19:**
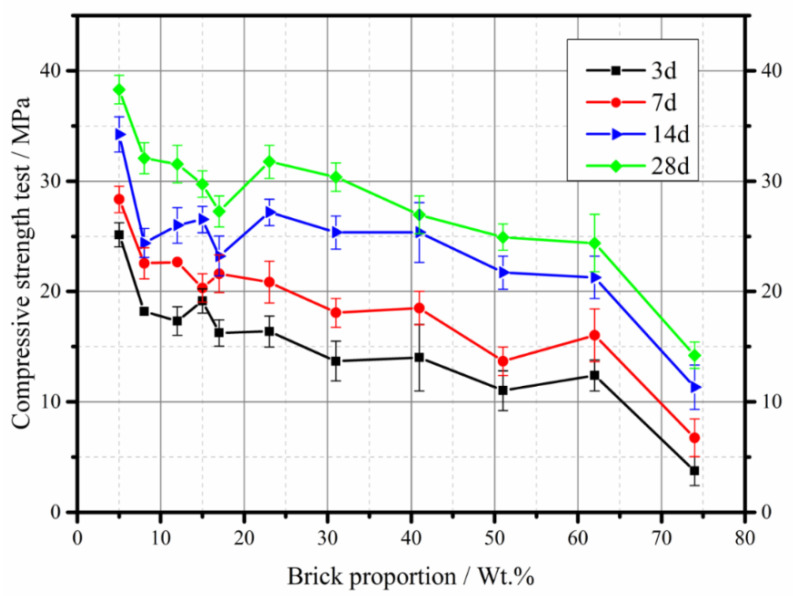
Compressive test of concrete mixture with different brick proportion of recycled materials.

**Figure 20:**
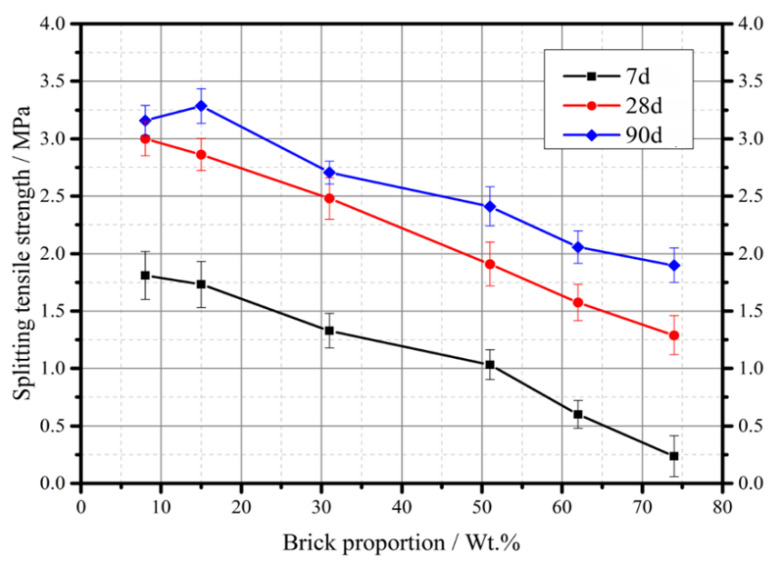
Splitting tensile test of concrete mixture with different brick proportion of recycled materials.

The concrete mixtures were prepared for small precast concrete curbs of highway construction, such as curbs. For this reason, compressive and splitting tensile tests were carried out to analyze the influence of brick ratio to mixture properties. Figure 19 and Figure 20 show the compressive strength and splitting tensile strength of concrete with different replacement ratio of brick. It should be noted that there were two values without standard deviation in Figure 19 because the samples broke during the curing period. The brick aggregates mixed in the concrete were recycled from the CDW stationary plant in Figure 15, with the particle sizes distribution of 9.5–19 mm and 19–31.5 mm. The compressive strength decreased with increase in brick proportions for the curing days of 3, 7, 14, and 28. It is noted that there was a fluctuation of compressive strength value in the brick proportion of 15 wt.%. Referring to the crushing value and stability tests in Figure 8 and Figure 9, the indexes varied dramatically in the brick proportion range between 10 wt.% and 20 wt.%. As a result of the brick ration in recycled aggregates separated less than 15 wt.%, the quality of mixed recycled aggregates was improved not only on the level of concrete manufacturing but also upgraded from subbase to base mixture application. The highest decrease in compressive strength was recorded at the brick proportion greater than 60%. The large decrease in compressive strength was also recorded from the brick proportion ranging from 41% to 62%. It is shown that the compressive strength significantly decreased as the brick proportion mixed in the concrete was larger than 41%. In general, the mixing of brick-mixed MRA played an unfavorable role in the compressive strength of concrete. The splitting tensile strength was also adversely affected by the inclusion of brick-mixed MRA at curing times of 7, 28, and 90 days. The failures of the test samples were mostly due to the broken brick aggregates or the interface between the brick and the cement binder as shown in Figure 20. Although the level of mixed recycled aggregates was still below the pavement aggregates acquirement after treatment, the reductions were significant and very promising in concrete manufacturing.

An anomalous data point was observed in the brick proportion ranging from 12 to 17% in Figure 19. This may be due to the brick being crushed when compressed with natural aggregates [28]. The compressive strength behavior of the MRA concrete in this study could be closely related to the low-quality grade of the RCA and weak bonding between the MRA and cement paste. Brick fraction is an important factor for the degradation of concrete properties. Previous studies also proved the adverse effect of MRA on concrete properties [29,30]. However, the mixed concrete with a brick proportion of 15% to 31% was less affected and the impact decreased steadily. The compressive strength remained at acceptable values for C30 grade concrete or used for curb production [31,32].

The slight different trends of the compressive and splitting tensile strengths, such as a sharp decrease in break contents of 12% to 17% and 62% to 74%, might be partly explained by the difference in the brick aggregates crushing mode during the loading tests [28] as shown in Figure 19. During the compaction process of the test samples, the crushed brick tends to break into finer particles with high specific surface and residual unhydrated cement components [30]. The crushed fines trend to hydrate with the water to guarantee workability of concrete mixture during compaction.

**Figure 21:**
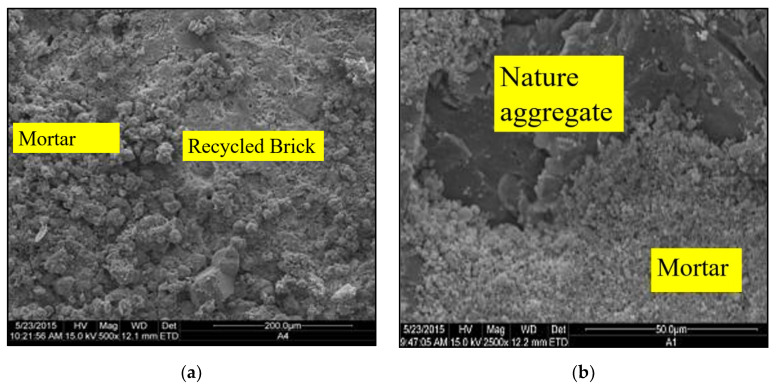
Microstructure analysis for CDW recycled concrete: (**a**) Bonding of mortar and recycled brick; (**b**) Bonding of mortar and nature aggregate.

It is worth noting that the recycled concrete aggregates contain both original particles and the mortar attached on them as shown in Figure 21. The new bonding mortar paste consists of two interfacial transition zones: one is the zone of the new mortar and recycled concrete aggregates (Figure 21a) and the other is the zone of the old mortar and original particle (Figure 21b). This generates weak regions in the microstructures of the new concrete particularly resulting in failure under compressive or bending loads. Therefore, this is another consideration to explain the decrease in fracture energy and flexural strength with increasing brick/MRA proportions.

Overall, brick separation and surface treatment technology proposed in this study achieved the following results.

- The brick fraction is problematic for construction and building materials because of its large water absorption ratio, weak strength, high flaky proportion, and low soundness stability.
- In general, it can be concluded that the brick fractions can be reduced from 45 wt.%~55 wt.% to less than 15 wt.% after processed by brick separation technology adopted in this study. Moreover, this technology innovation was proved to be practical in a stationary plant with a production capacity of 50–70 tons/hour.
- Regarding the surface treatment method adopted for MRA, R values of MRA in range of 1.5 to 6.5 were gradually reduced to the range of 1.1 to 2.0. This indicates that the flaky structure content of MRA was notably reduced.
- Twenty-five to thirty percent of the RCA weakness and attached mortar were removed by using the brick separation and surface treatment method in the stationary CDW recycling plant.
- The quality of recycled aggregates was upgraded from level-3 to level-2 based on the standard for recycled concrete materials currently used in China. As the same time, the quality was upgraded from subbase material to base material of highway construction based on standard for pavement materials in China.
- The compressive and splitting tensile properties were enhanced with low proportion of the brick fraction. The upgraded recycled materials were applied in base layer and small precast concrete curbs in a 113 km highway construction. Considerable economic and environment benefits were obtained by the CDW recycling project equipped with brick separation and surface treatment method proposed in this study.

## 4. Conclusions

In general terms, it can be concluded that the brick and attached mortar fractions in CDW were considerably reduced or eliminated by using the brick separation and surface treatment solution proposed in this study. The quality level of recycled aggregates was upgraded from subbase and foundation construction to base layer and precast concrete units manufacturing. The brick separation and surface treatment technology therefore constituents a promising solution allowing recycling plants to increase the value of the recycled CDW aggregates, especially when dealing with the mixed brick constituents of CDW.

Overall, brick separation technology achieves a large processing capacity in a practically low cost compare with existing gravity concentration, color, X-ray, near-infrared brick separation technologies carried out at laboratory-scale. However, the recycling purity for concrete aggregates seems to be challenging and still in a lower separation efficiency level.

As a result of the brick separation and surface treatment technologies, the quality of recycled aggregates was improved not only on the level of unwanted constituents (brick and attached mortar), but also obtained a better shape and surface characteristics. Although the recycled aggregates purity was still in a lower level, the reductions are significant and very practical when CDW recycling for a large-scale project, such as highway construction.

## Figures and Tables

**Table 1:** Chemical compositions of CDW aggregates and natural limestone.

Compound/Element (wt.%)	SiO_2_	Al_2_O_3_	Fe_2_O_3_	CaO	MgO	SO_3_	K_2_O	Others
Brick	69.22	11.29	8.64	3.87	1.42	-	-	2.21
Concrete	45.07	10.43	3.02	19.81	1.01	0.52	3.04	12.90
Mortar	38.52	12.32	3.49	25.41	1.72	0.23	2.07	15.20
Lime stone	0.82	0.31	0.94	55.72	0.34	0.02	0.13	41.31
Clay	75.15	9.56	3.18	0.96	0.71	-	3.73	4.16

**Table 2:** Recycled brick proportion of the concrete mixtures (kg/m^3^).

Brick Proportion in MRA	Cement	Flyash	Water	Fine Aggregates 0–4.75 mm	Natural Aggregates	Recycled Brick		Recycled Concrete		Additives
					4.75–9.5 mm	9.5–19 mm	19–31.5 mm	9.5–19 mm	19–31.5 mm	
5 wt.%	275	70	165	700	165	21	21	368	368	6.9
8 wt.%	275	70	165	700	165	29	29	356	356	6.9
12 wt.%	275	70	165	700	165	37	37	344	344	6.9
15 wt.%	275	70	165	700	165	45	45	333	333	6.9
17 wt.%	275	70	165	700	165	62	62	309	309	6.9
23 wt.%	275	70	165	700	165	83	83	274	274	6.9
31 wt.%	275	70	165	700	165	110	110	235	235	6.9
41 wt.%	275	70	165	700	165	138	138	196	196	6.9
51 wt.%	275	70	165	700	165	165	165	156	156	6.9
62 wt.%	275	70	165	700	165	193	193	117	117	6.9
74 wt.%	275	70	165	700	165	220	220	78	78	6.9
